# Event and Comment

**Published:** 1922-10

**Authors:** 


### DENTAL REGISTER

THE MAGAZINE OF DENTISTRY

Vol. LXXVI | OCTOBER, 1922 | No. 10

### EVENT AND COMMENT

### ANNUAL MEETING OF THE NATIONAL DENTAL ASSOCIATION.

(Now the American Dental Association.)

The Twenty-sixth Annual Meeting was held in the City of Los Angeles July 17th to 21st inclusive.

The attendance was 2,500 dentists and as many more visiting dentists and non-members. The weather was admirable in every particular, and the dentists of the Pacific Coast and the people of Los Angeles did everything they could to make the dentists have a good time, and see the many interesting sights of that part of the country.

The program under the direction of President Hartzell and his able and wise officers did nobly in carrying out the work as scheduled. As organized, the most important branch of the program was delegated to education in general. The Public Health Exhibit was centered on instruction, as was shown in such works as were given to exhibits of information tending to show how the inorganic salts might be utilized to produce higher grade of calcified tooth structures; and then how better information could be given the public in regard to the care of the teeth, etc.

Papers and studies intending to prevent dental caries and peridental diseases were important contributions, as were important studies on diet of children. An unusual emphasis was given to prophylaxis and endeavors to secure better stable tissue structures as preventive aids. In fact, some one suggested that the entire meeting had resolved itself into a great post graduate course of instruction in the science and art of healing and conservative dentistry. It is perhaps just as well to say that President Hartzell purposely made this impression by his slogan that said, ''Dentistry can add ten years to human life.''

It is of more than passing interest that the name National Dental Association was changed to The American Dental Association. This is a revision to the name which characterized this journal until it was changed to meet the ideas of the southern dentists a dozen years ago. We are glad the name has been restored, as it certainly is more appropriate and more significantly titles this great body of unified workers in a common cause. The new name has been incorporated in the State of Illinois, and it is now legally established as well as in harmony with the American Medical Association.

Another change was made in the adoption of a new item in our Code of Ethics. It is now unprofessional for dentists to pay or accept commissions, on fees for professional services, or for radiograms, or prescriptions, or other articles supplied to patients by pharmacists or others. Such actions renders one liable to expulsion from his dental society.

There was a strong effort made to bring about sufficient sentiment to get our state laws changed to permit reciprocal change of location for legally registered dentists in all states.

The favorable impression made by the members attending the meeting will surely make it practicable to bring the American Dental Association again to the Pacific Coast. The next annual meeting is scheduled for Cleveland, though why Detroit should be left out again, we do not quite understand. It is proposed that the ''Journal'' be printed semi-monthly and priced at $3 per year. Dr. John P. Buckley was elected president; and Dr. W. A. Giffen was made president-elect.

The following items in the report of the secretary-treasurer may be of interest as indicating the activities and the prosperous condition of the Association. These are only a brief synopsis of the more complete report, which might well be studied in more detail:

| | |
|---|---|
| Finances— | |
| Current Assets | $ 36,266.51 |
| Funded Assets | 167,509.52 |
| Fixed Assets | 13,469.91 |
| Making Total of | $217,245.94 |
| Some Important Assets are as follows— | |
| Relief Fund | $ 78,594.54 |
| Research Fund | 34,017.52 |
| Journal Reserve | 54,897.46 |
| General Fund | 33,346.60 |
| Receipts from— | |
| Advertising | $ 58,847.06 |
| Journal Subscribers | 27,704.96 |
| Membership Dues | 27,309.00 |
| Incidental | 1,461.21 |
| Total | $115,322.23 |
| Expenses— | |
| Publishing Journal | $ 60,270.59 |
| Secretary Department | 23,782.93 |
| Annual Session | 4,940.65 |
| President's Office | 1,112.67 |
| Treasurer | 647.75 |
| Hygiene | 794.46 |
| Committees | 1,283.80 |
| Trustees | 851.69 |
| Total | $ 93,684.50 |

**Index of Periodical Dental Literature for 1921**

In September of this year, 1922, there has been issued the first of a series of annual publications under this title, which it is proposed to print annually for the purpose of keeping our dental literature up to date in its records, and thus make it always up to date and readily accessible for everyone for the current year. This new publication has just come to hand and it records the indexing for 1921. It is printed in type and general form such as the two volumes already published. The plan is that we shall have printed each year all the material that is developed for each year; and it is to be published at the end of each year and it will be printed in the same form as the regular volumes have been heretofore done, with the exception that at the end of each five years the five volumes that are annually printed shall be re-edited and reprinted into larger volumes so that they can be more substantially printed and bound for permanent record.

The scheme is ideal, as it will not require one to look up old references when looking up data for new work, except, of course, for any unpublished material for the current years. In the past it has been almost impracticable to get material when wanted without much trouble and expense. The index will undoubtedly stimulate all such as may wish to engage in original research, because at little trouble he will have all past records readily available when wanted. It has been frequently charged that the dental profession has done very little for our literature. Undoubtedly this is largely due to the fact that there has been little to help the profession keep up with related literary endeavors. It is to be hoped we shall now be encouraged to the promotion of more extensive reading, and so helped to get the habit of thinking and recording scientific and technical ideas. If we should develop a literary habit, if it is only in thinking, and have a reasonable encouragement to write, we shall soon develope an ample literary output, even though its quality may not be of such a character that we may esteem it highly.

We think this is ample cause for congratulation, and we hope the results will justify the expense and effort made to bring this idea about.

